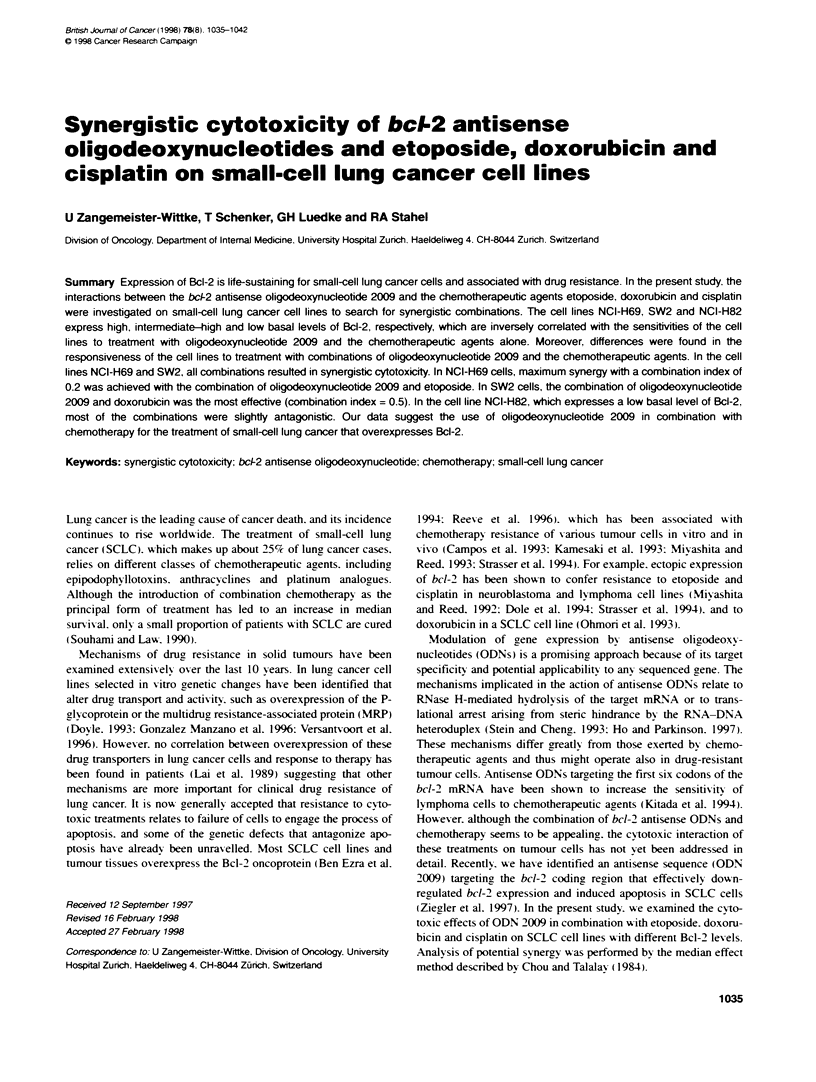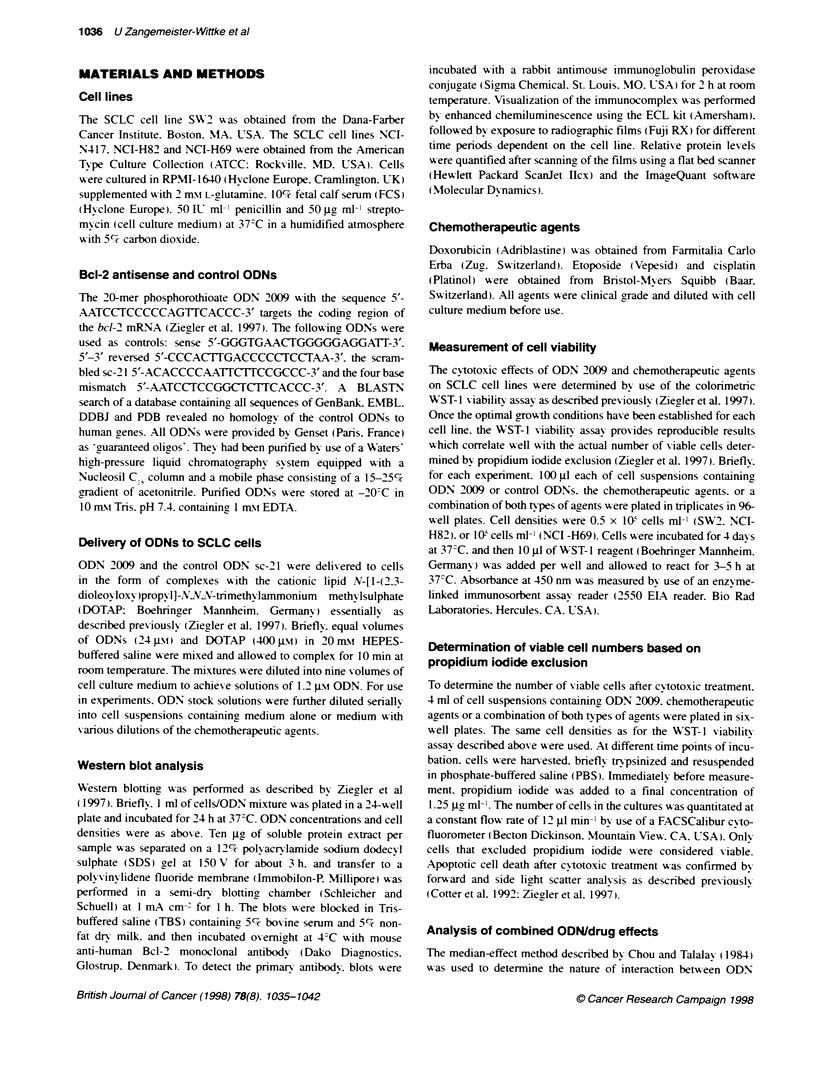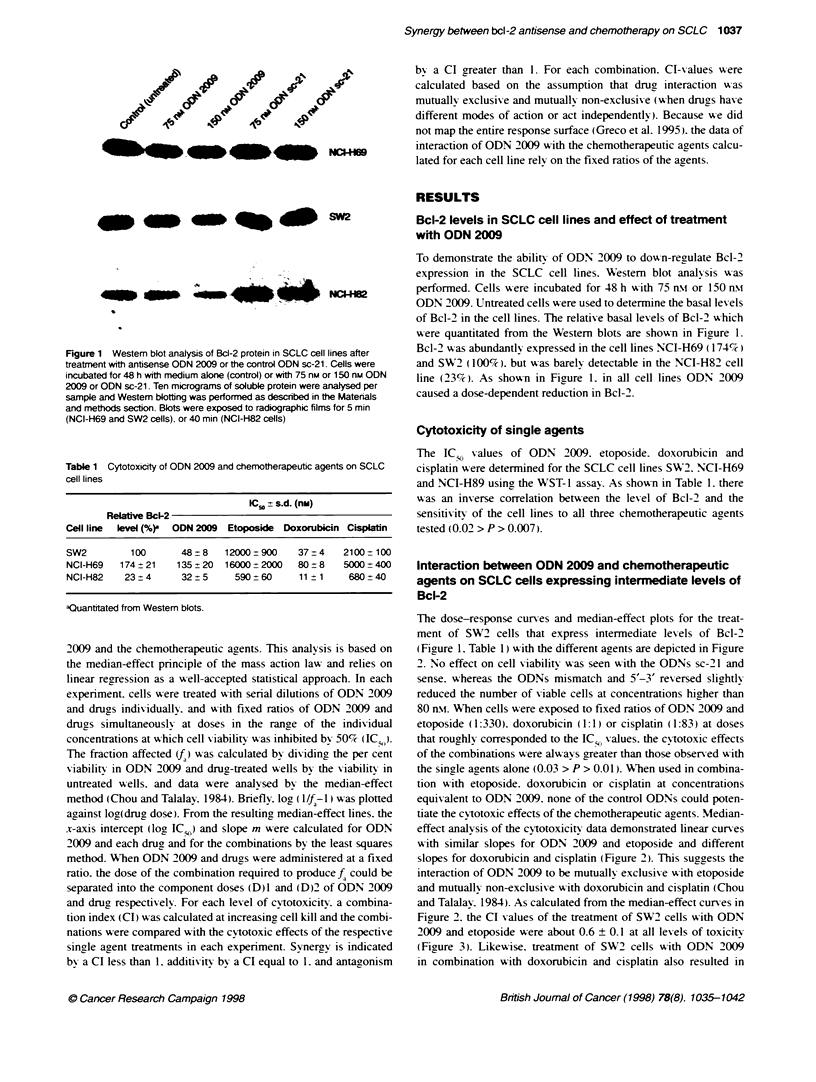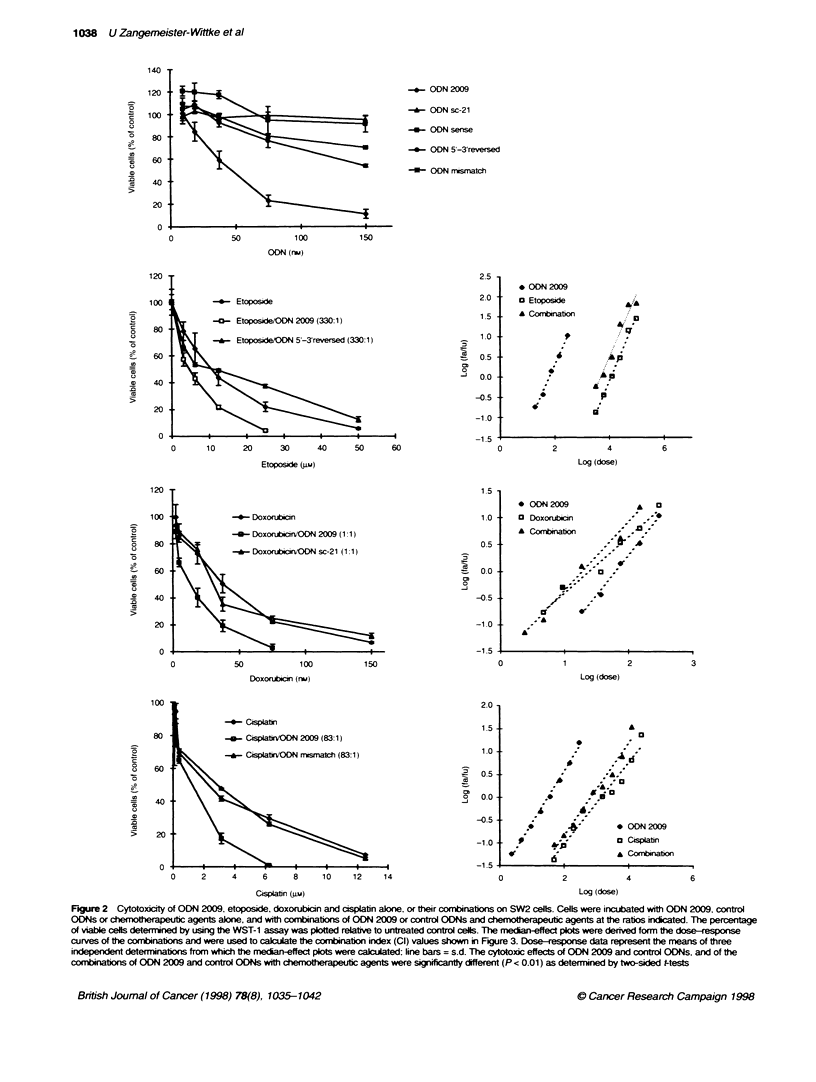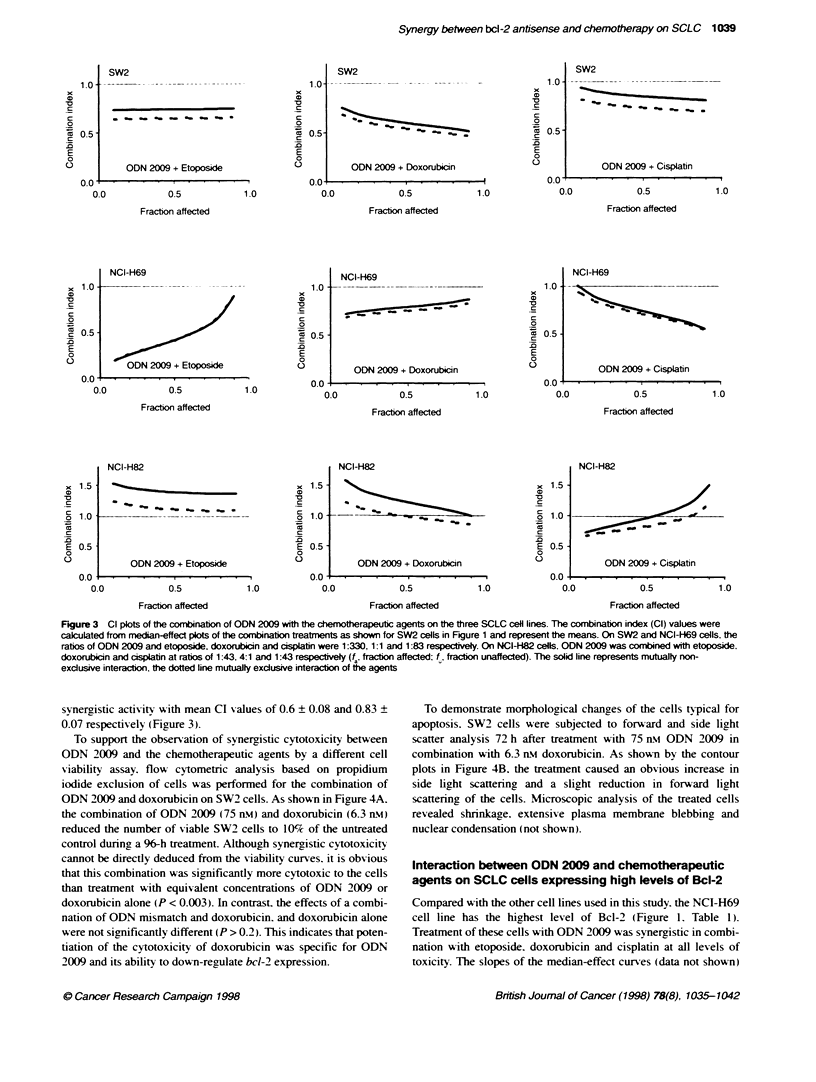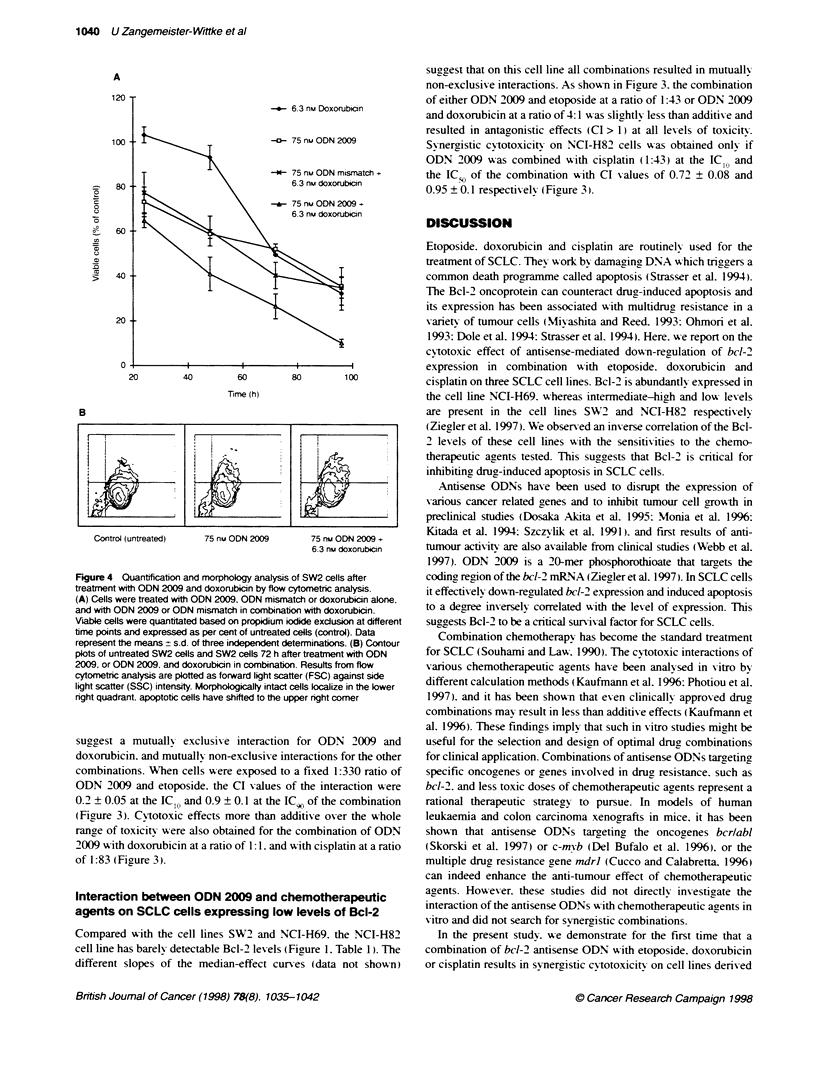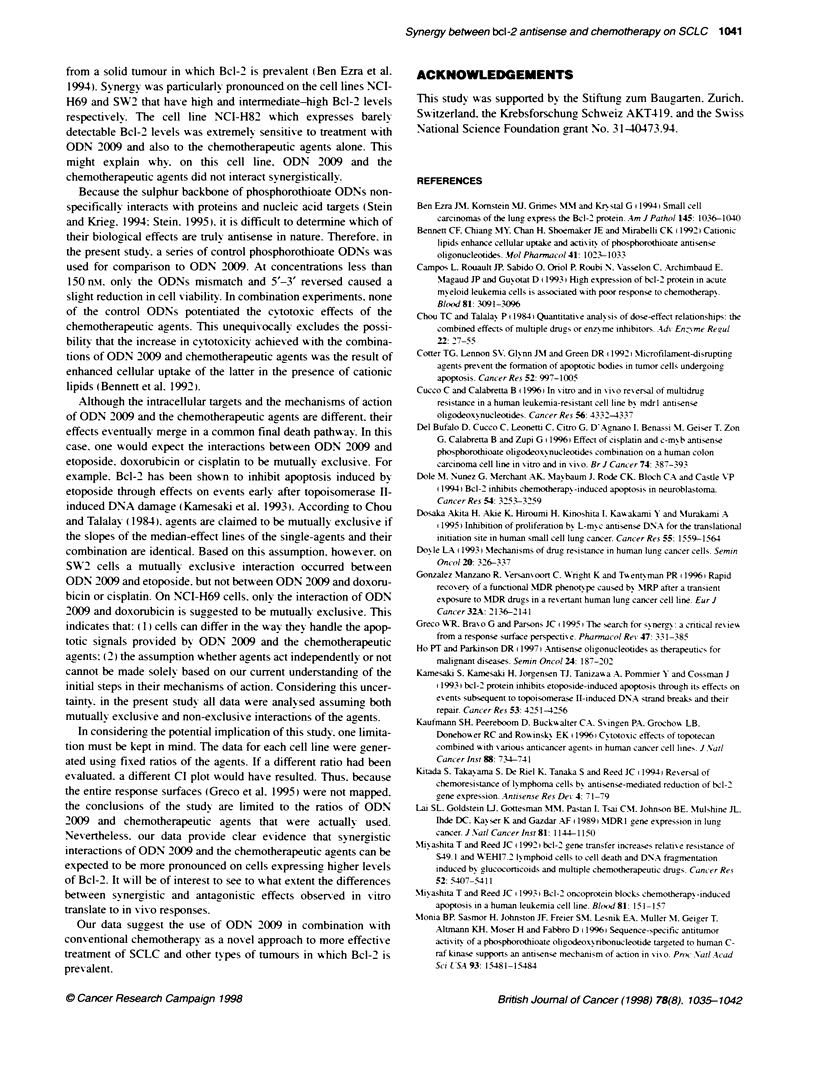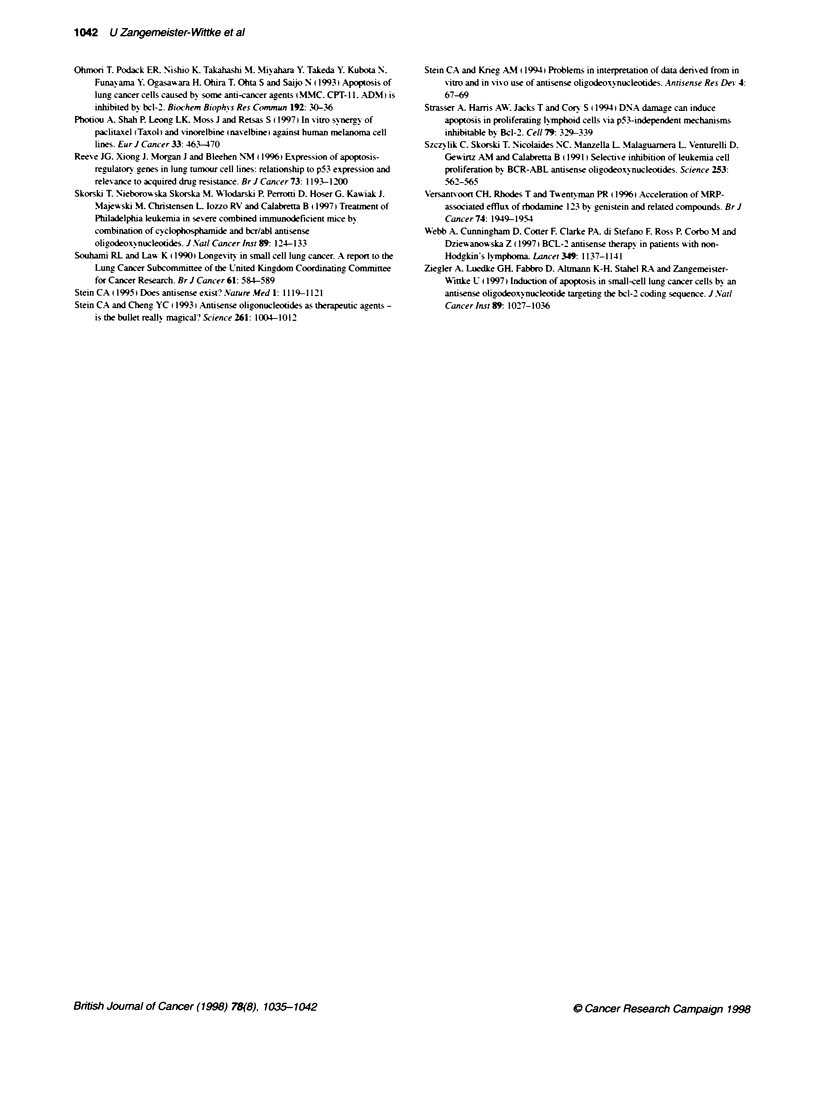# Synergistic cytotoxicity of bcl-2 antisense oligodeoxynucleotides and etoposide, doxorubicin and cisplatin on small-cell lung cancer cell lines.

**DOI:** 10.1038/bjc.1998.624

**Published:** 1998-10

**Authors:** U. Zangemeister-Wittke, T. Schenker, G. H. Luedke, R. A. Stahel

**Affiliations:** Department of Internal Medicine, University Hospital Zurich, Switzerland.

## Abstract

**Images:**


British Journal of Cancer (1998) 78(8). 1035–1042
© 1998 Cancer Research Campaign

### Synergistic cytotoxicity of *bcl*-2 antisense oligodeoxynucleotides and etoposide, doxorubicin and cisplatin on small-cell lung cancer cell lines

**U Zangemeister-Wittke, T Schenker, GH Luedke and RA Stahel**

Division of Oncology. Department of Internal Medicine. University Hospital Zurich. Haeldeliweg 4. CH-8044 Zurich. Switzerland

**Summary** Expression of Bcl-2 is life-sustaining for small-cell lung cancer cells and associated with drug resistance. In the present study, the interactions between the *bcl*-2 antisense oligodeoxynucleotide 2009 and the chemotherapeutic agents etoposide, doxorubicin and cisplatin were investigated on small-cell lung cancer cell lines to search for synergistic combinations. The cell lines NCI-H69, SW2 and NCI-H82 express high, intermediate–high and low basal levels of Bcl-2, respectively, which are inversely correlated with the sensitivities of the cell lines to treatment with oligodeoxynucleotide 2009 and the chemotherapeutic agents alone. Moreover, differences were found in the responsiveness of the cell lines to treatment with combinations of oligodeoxynucleotide 2009 and the chemotherapeutic agents. In the cell lines NCI-H69 and SW2, all combinations resulted in synergistic cytotoxicity. In NCI-H69 cells, maximum synergy with a combination index of 0.2 was achieved with the combination of oligodeoxynucleotide 2009 and etoposide. In SW2 cells, the combination of oligodeoxynucleotide 2009 and doxorubicin was the most effective (combination index = 0.5). In the cell line NCI-H82, which expresses a low basal level of Bcl-2, most of the combinations were slightly antagonistic. Our data suggest the use of oligodeoxynucleotide 2009 in combination with chemotherapy for the treatment of small-cell lung cancer that overexpresses Bcl-2.

**Keywords:** synergistic cytotoxicity; *bcl*-2 antisense oligodeoxynucleotide; chemotherapy; small-cell lung cancer

Lung cancer is the leading cause of cancer death, and its incidence continues to rise worldwide. The treatment of small-cell lung cancer (SCLC), which makes up about 25% of lung cancer cases, relies on different classes of chemotherapeutic agents, including epipodophyllotoxins, anthracyclines and platinum analogues. Although the introduction of combination chemotherapy as the principal form of treatment has led to an increase in median survival, only a small proportion of patients with SCLC are cured (Souhami and Law, 1990).

Mechanisms of drug resistance in solid tumours have been examined extensively over the last 10 years. In lung cancer cell lines selected in vitro genetic changes have been identified that alter drug transport and activity, such as overexpression of the P-glycoprotein or the multidrug resistance-associated protein (MRP) (Doyle, 1993; Gonzalez Manzano et al, 1996; Versantvoort et al, 1996). However, no correlation between overexpression of these drug transporters in lung cancer cells and response to therapy has been found in patients (Lai et al, 1989) suggesting that other mechanisms are more important for clinical drug resistance of lung cancer. It is now generally accepted that resistance to cytotoxic treatments relates to failure of cells to engage the process of apoptosis, and some of the genetic defects that antagonize apoptosis have already been unravelled. Most SCLC cell lines and tumour tissues overexpress the Bcl-2 oncoprotein (Ben Ezra et al, 1994; Reeve et al, 1996), which has been associated with chemotherapy resistance of various tumour cells in vitro and in vivo (Campos et al, 1993; Kamesaki et al, 1993; Miyashita and Reed, 1993; Strasser et al, 1994). For example, ectopic expression of *bcl*-2 has been shown to confer resistance to etoposide and cisplatin in neuroblastoma and lymphoma cell lines (Miyashita and Reed, 1992; Dole et al, 1994; Strasser et al, 1994), and to doxorubicin in a SCLC cell line (Ohmori et al, 1993).

Modulation of gene expression by antisense oligodeoxynucleotides (ODNs) is a promising approach because of its target specificity and potential applicability to any sequenced gene. The mechanisms implicated in the action of antisense ODNs relate to RNase H-mediated hydrolysis of the target mRNA or to translational arrest arising from steric hindrance by the RNA–DNA heteroduplex (Stein and Cheng, 1993; Ho and Parkinson, 1997). These mechanisms differ greatly from those exerted by chemotherapeutic agents and thus might operate also in drug-resistant tumour cells. Antisense ODNs targeting the first six codons of the *bcl*-2 mRNA have been shown to increase the sensitivity of lymphoma cells to chemotherapeutic agents (Kitada et al, 1994). However, although the combination of *bcl*-2 antisense ODNs and chemotherapy seems to be appealing, the cytotoxic interaction of these treatments on tumour cells has not yet been addressed in detail. Recently, we have identified an antisense sequence (ODN 2009) targeting the *bcl*-2 coding region that effectively down-regulated *bcl*-2 expression and induced apoptosis in SCLC cells (Ziegler et al, 1997). In the present study, we examined the cytotoxic effects of ODN 2009 in combination with etoposide, doxorubicin and cisplatin on SCLC cell lines with different Bcl-2 levels. Analysis of potential synergy was performed by the median effect method described by Chou and Talalay (1984).

*Received 12 September 1997*
*Revised 16 February 1998*
*Accepted 27 February 1998*

*Correspondence to:* U Zangemeister-Wittke, Division of Oncology, University Hospital Zurich, Haeldeliweg 4, CH-8044 Zürich, Switzerland

### MATERIALS AND METHODS

### Cell lines

The SCLC cell line SW2 was obtained from the Dana-Farber Cancer Institute, Boston, MA, USA. The SCLC cell lines NCI-N417, NCI-H82 and NCI-H69 were obtained from the American Type Culture Collection (ATCC; Rockville, MD, USA). Cells were cultured in RPMI-1640 (Hyclone Europe, Cramlington, UK) supplemented with 2 mM L-glutamine, 10% fetal calf serum (FCS) (Hyclone Europe), 50 IU ml$^{-1}$ penicillin and 50 µg ml$^{-1}$ streptomycin (cell culture medium) at 37°C in a humidified atmosphere with 5% carbon dioxide.

### Bcl-2 antisense and control ODNs

The 20-mer phosphorothioate ODN 2009 with the sequence 5′-AATCCTCCCCCAGTTCACCC-3′ targets the coding region of the *bcl*-2 mRNA (Ziegler et al, 1997). The following ODNs were used as controls: sense 5′-GGGTGAACTGGGGGAGGATT-3′, 5′–3′ reversed 5′-CCCACTTGACCCCCTCCTAA-3′, the scrambled sc-21 5′-ACACCCCAATTCTTCCGCCC-3′ and the four base mismatch 5′-AATCCTCCGGCTCTTCACCC-3′. A BLASTN search of a database containing all sequences of GenBank, EMBL, DDBJ and PDB revealed no homology of the control ODNs to human genes. All ODNs were provided by Genset (Paris, France) as 'guaranteed oligos'. They had been purified by use of a Waters' high-pressure liquid chromatography system equipped with a Nucleosil $C_{18}$ column and a mobile phase consisting of a 15–25% gradient of acetonitrile. Purified ODNs were stored at –20°C in 10 mM Tris, pH 7.4, containing 1 mM EDTA.

### Delivery of ODNs to SCLC cells

ODN 2009 and the control ODN sc-21 were delivered to cells in the form of complexes with the cationic lipid *N*-[1-(2,3-dioleoyloxy)propyl]-*N,N,N*-trimethylammonium methylsulphate (DOTAP; Boehringer Mannheim, Germany) essentially as described previously (Ziegler et al, 1997). Briefly, equal volumes of ODNs (24 µM) and DOTAP (400 µM) in 20 mM HEPES-buffered saline were mixed and allowed to complex for 10 min at room temperature. The mixtures were diluted into nine volumes of cell culture medium to achieve solutions of 1.2 µM ODN. For use in experiments, ODN stock solutions were further diluted serially into cell suspensions containing medium alone or medium with various dilutions of the chemotherapeutic agents.

### Western blot analysis

Western blotting was performed as described by Ziegler et al (1997). Briefly, 1 ml of cells/ODN mixture was plated in a 24-well plate and incubated for 24 h at 37°C. ODN concentrations and cell densities were as above. Ten µg of soluble protein extract per sample was separated on a 12% polyacrylamide sodium dodecyl sulphate (SDS) gel at 150 V for about 3 h, and transfer to a polyvinylidene fluoride membrane (Immobilon-P, Millipore) was performed in a semi-dry blotting chamber (Schleicher and Schuell) at 1 mA cm$^{-2}$ for 1 h. The blots were blocked in Tris-buffered saline (TBS) containing 5% bovine serum and 5% non-fat dry milk, and then incubated overnight at 4°C with mouse anti-human Bcl-2 monoclonal antibody (Dako Diagnostics, Glostrup, Denmark). To detect the primary antibody, blots were incubated with a rabbit antimouse immunoglobulin peroxidase conjugate (Sigma Chemical, St. Louis, MO, USA) for 2 h at room temperature. Visualization of the immunocomplex was performed by enhanced chemiluminescence using the ECL kit (Amersham), followed by exposure to radiographic films (Fuji RX) for different time periods dependent on the cell line. Relative protein levels were quantified after scanning of the films using a flat bed scanner (Hewlett Packard ScanJet IIcx) and the ImageQuant software (Molecular Dynamics).

### Chemotherapeutic agents

Doxorubicin (Adriblastine) was obtained from Farmitalia Carlo Erba (Zug, Switzerland). Etoposide (Vepesid) and cisplatin (Platinol) were obtained from Bristol-Myers Squibb (Baar, Switzerland). All agents were clinical grade and diluted with cell culture medium before use.

### Measurement of cell viability

The cytotoxic effects of ODN 2009 and chemotherapeutic agents on SCLC cell lines were determined by use of the colorimetric WST-1 viability assay as described previously (Ziegler et al, 1997). Once the optimal growth conditions have been established for each cell line, the WST-1 viability assay provides reproducible results which correlate well with the actual number of viable cells determined by propidium iodide exclusion (Ziegler et al, 1997). Briefly, for each experiment, 100 µl each of cell suspensions containing ODN 2009 or control ODNs, the chemotherapeutic agents, or a combination of both types of agents were plated in triplicates in 96-well plates. Cell densities were 0.5 × 10$^5$ cells ml$^{-1}$ (SW2, NCI-H82), or 10$^5$ cells ml$^{-1}$ (NCI-H69). Cells were incubated for 4 days at 37°C, and then 10 µl of WST-1 reagent (Boehringer Mannheim, Germany) was added per well and allowed to react for 3–5 h at 37°C. Absorbance at 450 nm was measured by use of an enzyme-linked immunosorbent assay reader (2550 EIA reader, Bio Rad Laboratories, Hercules, CA, USA).

### Determination of viable cell numbers based on propidium iodide exclusion

To determine the number of viable cells after cytotoxic treatment, 4 ml of cell suspensions containing ODN 2009, chemotherapeutic agents or a combination of both types of agents were plated in six-well plates. The same cell densities as for the WST-1 viability assay described above were used. At different time points of incubation, cells were harvested, briefly trypsinized and resuspended in phosphate-buffered saline (PBS). Immediately before measurement, propidium iodide was added to a final concentration of 1.25 µg ml$^{-1}$. The number of cells in the cultures was quantitated at a constant flow rate of 12 µl min$^{-1}$ by use of a FACSCalibur cytofluorometer (Becton Dickinson, Mountain View, CA, USA). Only cells that excluded propidium iodide were considered viable. Apoptotic cell death after cytotoxic treatment was confirmed by forward and side light scatter analysis as described previously (Cotter et al, 1992; Ziegler et al, 1997).

### Analysis of combined ODN/drug effects

The median-effect method described by Chou and Talalay (1984) was used to determine the nature of interaction between ODN

© Cancer Research Campaign 1998

**Figure 1** Western blot analysis of Bcl-2 protein in SCLC cell lines after treatment with antisense ODN 2009 or the control ODN sc-21. Cells were incubated for 48 h with medium alone (control) or with 75 nM or 150 nM ODN 2009 or ODN sc-21. Ten micrograms of soluble protein were analysed per sample and Western blotting was performed as described in the Materials and methods section. Blots were exposed to radiographic films for 5 min (NCI-H69 and SW2 cells), or 40 min (NCI-H82 cells)

**Table 1** Cytotoxicity of ODN 2009 and chemotherapeutic agents on SCLC cell lines

| | | $IC_{50}$ ± s.d. (nM) | | | |
|---|---|---|---|---|---|
| **Cell line** | **Relative Bcl-2 level (%)[a]** | **ODN 2009** | **Etoposide** | **Doxorubicin** | **Cisplatin** |
| SW2 | 100 | 48 ± 8 | 12000 ± 900 | 37 ± 4 | 2100 ± 100 |
| NCI-H69 | 174 ± 21 | 135 ± 20 | 16000 ± 2000 | 80 ± 8 | 5000 ± 400 |
| NCI-H82 | 23 ± 4 | 32 ± 5 | 590 ± 60 | 11 ± 1 | 680 ± 40 |

[a]Quantitated from Western blots.

2009 and the chemotherapeutic agents. This analysis is based on the median-effect principle of the mass action law and relies on linear regression as a well-accepted statistical approach. In each experiment, cells were treated with serial dilutions of ODN 2009 and drugs individually, and with fixed ratios of ODN 2009 and drugs simultaneously at doses in the range of the individual concentrations at which cell viability was inhibited by 50% ($IC_{50}$). The fraction affected ($f_a$) was calculated by dividing the per cent viability in ODN 2009 and drug-treated wells by the viability in untreated wells, and data were analysed by the median-effect method (Chou and Talalay, 1984). Briefly, log ($1/f_a-1$) was plotted against log(drug dose). From the resulting median-effect lines, the $x$-axis intercept (log $IC_{50}$) and slope $m$ were calculated for ODN 2009 and each drug and for the combinations by the least squares method. When ODN 2009 and drugs were administered at a fixed ratio, the dose of the combination required to produce $f_a$ could be separated into the component doses (D)1 and (D)2 of ODN 2009 and drug respectively. For each level of cytotoxicity, a combination index (CI) was calculated at increasing cell kill and the combinations were compared with the cytotoxic effects of the respective single agent treatments in each experiment. Synergy is indicated by a CI less than 1, additivity by a CI equal to 1, and antagonism by a CI greater than 1. For each combination, CI-values were calculated based on the assumption that drug interaction was mutually exclusive and mutually non-exclusive (when drugs have different modes of action or act independently). Because we did not map the entire response surface (Greco et al, 1995), the data of interaction of ODN 2009 with the chemotherapeutic agents calculated for each cell line rely on the fixed ratios of the agents.

### RESULTS

### Bcl-2 levels in SCLC cell lines and effect of treatment with ODN 2009

To demonstrate the ability of ODN 2009 to down-regulate Bcl-2 expression in the SCLC cell lines, Western blot analysis was performed. Cells were incubated for 48 h with 75 nM or 150 nM ODN 2009. Untreated cells were used to determine the basal levels of Bcl-2 in the cell lines. The relative basal levels of Bcl-2 which were quantitated from the Western blots are shown in Figure 1. Bcl-2 was abundantly expressed in the cell lines NCI-H69 (174%) and SW2 (100%), but was barely detectable in the NCI-H82 cell line (23%). As shown in Figure 1, in all cell lines ODN 2009 caused a dose-dependent reduction in Bcl-2.

### Cytotoxicity of single agents

The $IC_{50}$ values of ODN 2009, etoposide, doxorubicin and cisplatin were determined for the SCLC cell lines SW2, NCI-H69 and NCI-H89 using the WST-1 assay. As shown in Table 1, there was an inverse correlation between the level of Bcl-2 and the sensitivity of the cell lines to all three chemotherapeutic agents tested ($0.02 > P > 0.007$).

### Interaction between ODN 2009 and chemotherapeutic agents on SCLC cells expressing intermediate levels of Bcl-2

The dose–response curves and median-effect plots for the treatment of SW2 cells that express intermediate levels of Bcl-2 (Figure 1, Table 1) with the different agents are depicted in Figure 2. No effect on cell viability was seen with the ODNs sc-21 and sense, whereas the ODNs mismatch and 5′–3′ reversed slightly reduced the number of viable cells at concentrations higher than 80 nM. When cells were exposed to fixed ratios of ODN 2009 and etoposide (1:330), doxorubicin (1:1) or cisplatin (1:83) at doses that roughly corresponded to the $IC_{50}$ values, the cytotoxic effects of the combinations were always greater than those observed with the single agents alone ($0.03 > P > 0.01$). When used in combination with etoposide, doxorubicin or cisplatin at concentrations equivalent to ODN 2009, none of the control ODNs could potentiate the cytotoxic effects of the chemotherapeutic agents. Median-effect analysis of the cytotoxicity data demonstrated linear curves with similar slopes for ODN 2009 and etoposide and different slopes for doxorubicin and cisplatin (Figure 2). This suggests the interaction of ODN 2009 to be mutually exclusive with etoposide and mutually non-exclusive with doxorubicin and cisplatin (Chou and Talalay, 1984). As calculated from the median-effect curves in Figure 2, the CI values of the treatment of SW2 cells with ODN 2009 and etoposide were about 0.6 ± 0.1 at all levels of toxicity (Figure 3). Likewise, treatment of SW2 cells with ODN 2009 in combination with doxorubicin and cisplatin also resulted in

© Cancer Research Campaign 1998

**Figure 2** Cytotoxicity of ODN 2009, etoposide, doxorubicin and cisplatin alone, or their combinations on SW2 cells. Cells were incubated with ODN 2009, control ODNs or chemotherapeutic agents alone, and with combinations of ODN 2009 or control ODNs and chemotherapeutic agents at the ratios indicated. The percentage of viable cells determined by using the WST-1 assay was plotted relative to untreated control cells. The median-effect plots were derived form the dose–response curves of the combinations and were used to calculate the combination index (CI) values shown in Figure 3. Dose–response data represent the means of three independent determinations from which the median-effect plots were calculated; line bars = s.d. The cytotoxic effects of ODN 2009 and control ODNs, and of the combinations of ODN 2009 and control ODNs with chemotherapeutic agents were significantly different ($P < 0.01$) as determined by two-sided $t$-tests

© Cancer Research Campaign 1998

**Figure 3** CI plots of the combination of ODN 2009 with the chemotherapeutic agents on the three SCLC cell lines. The combination index (CI) values were calculated from median-effect plots of the combination treatments as shown for SW2 cells in Figure 1 and represent the means. On SW2 and NCI-H69 cells, the ratios of ODN 2009 and etoposide, doxorubicin and cisplatin were 1:330, 1:1 and 1:83 respectively. On NCI-H82 cells, ODN 2009 was combined with etoposide, doxorubicin and cisplatin at ratios of 1:43, 4:1 and 1:43 respectively ($f_a$, fraction affected; $f_u$, fraction unaffected). The solid line represents mutually non-exclusive interaction, the dotted line mutually exclusive interaction of the agents synergistic activity with mean CI values of 0.6 ± 0.08 and 0.83 ± 0.07 respectively (Figure 3).

To support the observation of synergistic cytotoxicity between ODN 2009 and the chemotherapeutic agents by a different cell viability assay, flow cytometric analysis based on propidium iodide exclusion of cells was performed for the combination of ODN 2009 and doxorubicin on SW2 cells. As shown in Figure 4A, the combination of ODN 2009 (75 nM) and doxorubicin (6.3 nM) reduced the number of viable SW2 cells to 10% of the untreated control during a 96-h treatment. Although synergistic cytotoxicity cannot be directly deduced from the viability curves, it is obvious that this combination was significantly more cytotoxic to the cells than treatment with equivalent concentrations of ODN 2009 or doxorubicin alone ($P < 0.003$). In contrast, the effects of a combination of ODN mismatch and doxorubicin, and doxorubicin alone were not significantly different ($P > 0.2$). This indicates that potentiation of the cytotoxicity of doxorubicin was specific for ODN 2009 and its ability to down-regulate *bcl*-2 expression.

To demonstrate morphological changes of the cells typical for apoptosis, SW2 cells were subjected to forward and side light scatter analysis 72 h after treatment with 75 nM ODN 2009 in combination with 6.3 nM doxorubicin. As shown by the contour plots in Figure 4B, the treatment caused an obvious increase in side light scattering and a slight reduction in forward light scattering of the cells. Microscopic analysis of the treated cells revealed shrinkage, extensive plasma membrane blebbing and nuclear condensation (not shown).

### Interaction between ODN 2009 and chemotherapeutic agents on SCLC cells expressing high levels of Bcl-2

Compared with the other cell lines used in this study, the NCI-H69 cell line has the highest level of Bcl-2 (Figure 1, Table 1). Treatment of these cells with ODN 2009 was synergistic in combination with etoposide, doxorubicin and cisplatin at all levels of toxicity. The slopes of the median-effect curves (data not shown)

© Cancer Research Campaign 1998

**Figure 4** Quantification and morphology analysis of SW2 cells after treatment with ODN 2009 and doxorubicin by flow cytometric analysis. (**A**) Cells were treated with ODN 2009, ODN mismatch or doxorubicin alone, and with ODN 2009 or ODN mismatch in combination with doxorubicin. Viable cells were quantitated based on propidium iodide exclusion at different time points and expressed as per cent of untreated cells (control). Data represent the means ± s.d. of three independent determinations. (**B**) Contour plots of untreated SW2 cells and SW2 cells 72 h after treatment with ODN 2009, or ODN 2009, and doxorubicin in combination. Results from flow cytometric analysis are plotted as forward light scatter (FSC) against side light scatter (SSC) intensity. Morphologically intact cells localize in the lower right quadrant, apoptotic cells have shifted to the upper right corner suggest a mutually exclusive interaction for ODN 2009 and doxorubicin, and mutually non-exclusive interactions for the other combinations. When cells were exposed to a fixed 1:330 ratio of ODN 2009 and etoposide, the CI values of the interaction were 0.2 ± 0.05 at the $IC_{10}$ and 0.9 ± 0.1 at the $IC_{90}$ of the combination (Figure 3). Cytotoxic effects more than additive over the whole range of toxicity were also obtained for the combination of ODN 2009 with doxorubicin at a ratio of 1:1, and with cisplatin at a ratio of 1:83 (Figure 3).

### Interaction between ODN 2009 and chemotherapeutic agents on SCLC cells expressing low levels of Bcl-2

Compared with the cell lines SW2 and NCI-H69, the NCI-H82 cell line has barely detectable Bcl-2 levels (Figure 1, Table 1). The different slopes of the median-effect curves (data not shown) suggest that on this cell line all combinations resulted in mutually non-exclusive interactions. As shown in Figure 3, the combination of either ODN 2009 and etoposide at a ratio of 1:43 or ODN 2009 and doxorubicin at a ratio of 4:1 was slightly less than additive and resulted in antagonistic effects (CI > 1) at all levels of toxicity. Synergistic cytotoxicity on NCI-H82 cells was obtained only if ODN 2009 was combined with cisplatin (1:43) at the $IC_{10}$ and the $IC_{50}$ of the combination with CI values of 0.72 ± 0.08 and 0.95 ± 0.1 respectively (Figure 3).

### DISCUSSION

Etoposide, doxorubicin and cisplatin are routinely used for the treatment of SCLC. They work by damaging DNA which triggers a common death programme called apoptosis (Strasser et al, 1994). The Bcl-2 oncoprotein can counteract drug-induced apoptosis and its expression has been associated with multidrug resistance in a variety of tumour cells (Miyashita and Reed, 1993; Ohmori et al, 1993; Dole et al, 1994; Strasser et al, 1994). Here, we report on the cytotoxic effect of antisense-mediated down-regulation of *bcl-2* expression in combination with etoposide, doxorubicin and cisplatin on three SCLC cell lines. Bcl-2 is abundantly expressed in the cell line NCI-H69, whereas intermediate–high and low levels are present in the cell lines SW2 and NCI-H82 respectively (Ziegler et al, 1997). We observed an inverse correlation of the Bcl-2 levels of these cell lines with the sensitivities to the chemotherapeutic agents tested. This suggests that Bcl-2 is critical for inhibiting drug-induced apoptosis in SCLC cells.

Antisense ODNs have been used to disrupt the expression of various cancer related genes and to inhibit tumour cell growth in preclinical studies (Dosaka Akita et al, 1995; Monia et al, 1996; Kitada et al, 1994; Szczylik et al, 1991), and first results of anti-tumour activity are also available from clinical studies (Webb et al, 1997). ODN 2009 is a 20-mer phosphorothioate that targets the coding region of the *bcl-2* mRNA (Ziegler et al, 1997). In SCLC cells it effectively down-regulated *bcl-2* expression and induced apoptosis to a degree inversely correlated with the level of expression. This suggests Bcl-2 to be a critical survival factor for SCLC cells.

Combination chemotherapy has become the standard treatment for SCLC (Souhami and Law, 1990). The cytotoxic interactions of various chemotherapeutic agents have been analysed in vitro by different calculation methods (Kaufmann et al, 1996; Photiou et al, 1997), and it has been shown that even clinically approved drug combinations may result in less than additive effects (Kaufmann et al, 1996). These findings imply that such in vitro studies might be useful for the selection and design of optimal drug combinations for clinical application. Combinations of antisense ODNs targeting specific oncogenes or genes involved in drug resistance, such as *bcl-2*, and less toxic doses of chemotherapeutic agents represent a rational therapeutic strategy to pursue. In models of human leukaemia and colon carcinoma xenografts in mice, it has been shown that antisense ODNs targeting the oncogenes *bcr/abl* (Skorski et al, 1997) or *c-myb* (Del Bufalo et al, 1996), or the multiple drug resistance gene *mdr1* (Cucco and Calabretta, 1996) can indeed enhance the anti-tumour effect of chemotherapeutic agents. However, these studies did not directly investigate the interaction of the antisense ODNs with chemotherapeutic agents in vitro and did not search for synergistic combinations.

In the present study, we demonstrate for the first time that a combination of *bcl-2* antisense ODN with etoposide, doxorubicin or cisplatin results in synergistic cytotoxicity on cell lines derived

© Cancer Research Campaign 1998

from a solid tumour in which Bcl-2 is prevalent (Ben Ezra et al. 1994). Synergy was particularly pronounced on the cell lines NCI-H69 and SW2 that have high and intermediate–high Bcl-2 levels respectively. The cell line NCI-H82 which expresses barely detectable Bcl-2 levels was extremely sensitive to treatment with ODN 2009 and also to the chemotherapeutic agents alone. This might explain why, on this cell line, ODN 2009 and the chemotherapeutic agents did not interact synergistically.

Because the sulphur backbone of phosphorothioate ODNs non-specifically interacts with proteins and nucleic acid targets (Stein and Krieg, 1994; Stein, 1995), it is difficult to determine which of their biological effects are truly antisense in nature. Therefore, in the present study, a series of control phosphorothioate ODNs was used for comparison to ODN 2009. At concentrations less than 150 nM, only the ODNs mismatch and 5′–3′ reversed caused a slight reduction in cell viability. In combination experiments, none of the control ODNs potentiated the cytotoxic effects of the chemotherapeutic agents. This unequivocally excludes the possibility that the increase in cytotoxicity achieved with the combinations of ODN 2009 and chemotherapeutic agents was the result of enhanced cellular uptake of the latter in the presence of cationic lipids (Bennett et al, 1992).

Although the intracellular targets and the mechanisms of action of ODN 2009 and the chemotherapeutic agents are different, their effects eventually merge in a common final death pathway. In this case, one would expect the interactions between ODN 2009 and etoposide, doxorubicin or cisplatin to be mutually exclusive. For example, Bcl-2 has been shown to inhibit apoptosis induced by etoposide through effects on events early after topoisomerase II-induced DNA damage (Kamesaki et al, 1993). According to Chou and Talalay (1984), agents are claimed to be mutually exclusive if the slopes of the median-effect lines of the single-agents and their combination are identical. Based on this assumption, however, on SW2 cells a mutually exclusive interaction occurred between ODN 2009 and etoposide, but not between ODN 2009 and doxorubicin or cisplatin. On NCI-H69 cells, only the interaction of ODN 2009 and doxorubicin is suggested to be mutually exclusive. This indicates that: (1) cells can differ in the way they handle the apoptotic signals provided by ODN 2009 and the chemotherapeutic agents; (2) the assumption whether agents act independently or not cannot be made solely based on our current understanding of the initial steps in their mechanisms of action. Considering this uncertainty, in the present study all data were analysed assuming both mutually exclusive and non-exclusive interactions of the agents.

In considering the potential implication of this study, one limitation must be kept in mind. The data for each cell line were generated using fixed ratios of the agents. If a different ratio had been evaluated, a different CI plot would have resulted. Thus, because the entire response surfaces (Greco et al, 1995) were not mapped, the conclusions of the study are limited to the ratios of ODN 2009 and chemotherapeutic agents that were actually used. Nevertheless, our data provide clear evidence that synergistic interactions of ODN 2009 and the chemotherapeutic agents can be expected to be more pronounced on cells expressing higher levels of Bcl-2. It will be of interest to see to what extent the differences between synergistic and antagonistic effects observed in vitro translate to in vivo responses.

Our data suggest the use of ODN 2009 in combination with conventional chemotherapy as a novel approach to more effective treatment of SCLC and other types of tumours in which Bcl-2 is prevalent.

### ACKNOWLEDGEMENTS

This study was supported by the Stiftung zum Baugarten, Zurich, Switzerland, the Krebsforschung Schweiz AKT419, and the Swiss National Science Foundation grant No. 31-40473.94.

### REFERENCES

Ben Ezra JM, Kornstein MJ, Grimes MM and Krystal G (1994) Small cell carcinomas of the lung express the Bcl-2 protein. *Am J Pathol* **145**: 1036–1040

Bennett CF, Chiang MY, Chan H, Shoemaker JE and Mirabelli CK (1992) Cationic lipids enhance cellular uptake and activity of phosphorothioate antisense oligonucleotides. *Mol Pharmacol* **41**: 1023–1033

Campos L, Rouault JP, Sabido O, Oriol P, Roubi N, Vasselon C, Archimbaud E, Magaud JP and Guyotat D (1993) High expression of bcl-2 protein in acute myeloid leukemia cells is associated with poor response to chemotherapy. *Blood* **81**: 3091–3096

Chou TC and Talalay P (1984) Quantitative analysis of dose-effect relationships: the combined effects of multiple drugs or enzyme inhibitors. *Adv Enzyme Regul* **22**: 27–55

Cotter TG, Lennon SV, Glynn JM and Green DR (1992) Microfilament-disrupting agents prevent the formation of apoptotic bodies in tumor cells undergoing apoptosis. *Cancer Res* **52**: 997–1005

Cucco C and Calabretta B (1996) In vitro and in vivo reversal of multidrug resistance in a human leukemia-resistant cell line by mdr1 antisense oligodeoxynucleotides. *Cancer Res* **56**: 4332–4337

Del Bufalo D, Cucco C, Leonetti C, Citro G, D'Agnano I, Benassi M, Geiser T, Zon G, Calabretta B and Zupi G (1996) Effect of cisplatin and c-myb antisense phosphorothioate oligodeoxynucleotides combination on a human colon carcinoma cell line in vitro and in vivo. *Br J Cancer* **74**: 387–393

Dole M, Nunez G, Merchant AK, Maybaum J, Rode CK, Bloch CA and Castle VP (1994) Bcl-2 inhibits chemotherapy-induced apoptosis in neuroblastoma. *Cancer Res* **54**: 3253–3259

Dosaka-Akita H, Akie K, Hiroumi H, Kinoshita I, Kawakami Y and Murakami A (1995) Inhibition of proliferation by L-myc antisense DNA for the translational initiation site in human small cell lung cancer. *Cancer Res* **55**: 1559–1564

Doyle LA (1993) Mechanisms of drug resistance in human lung cancer cells. *Semin Oncol* **20**: 326–337

Gonzalez Manzano R, Versantvoort C, Wright K and Twentyman PR (1996) Rapid recovery of a functional MDR phenotype caused by MRP after a transient exposure to MDR drugs in a revertant human lung cancer cell line. *Eur J Cancer* **32A**: 2136–2141

Greco WR, Bravo G and Parsons JC (1995) The search for synergy: a critical review from a response surface perspective. *Pharmacol Rev* **47**: 331–385

Ho PT and Parkinson DR (1997) Antisense oligonucleotides as therapeutics for malignant diseases. *Semin Oncol* **24**: 187–202

Kamesaki S, Kamesaki H, Jorgensen TJ, Tanizawa A, Pommier Y and Cossman J (1993) bcl-2 protein inhibits etoposide-induced apoptosis through its effects on events subsequent to topoisomerase II-induced DNA strand breaks and their repair. *Cancer Res* **53**: 4251–4256

Kaufmann SH, Peereboom D, Buckwalter CA, Svingen PA, Grochow LB, Donehower RC and Rowinsky EK (1996) Cytotoxic effects of topotecan combined with various anticancer agents in human cancer cell lines. *J Natl Cancer Inst* **88**: 734–741

Kitada S, Takayama S, De Riel K, Tanaka S and Reed JC (1994) Reversal of chemoresistance of lymphoma cells by antisense-mediated reduction of bcl-2 gene expression. *Antisense Res Dev* **4**: 71–79

Lai SL, Goldstein LJ, Gottesman MM, Pastan I, Tsai CM, Johnson BE, Mulshine JL, Ihde DC, Kayser K and Gazdar AF (1989) MDR1 gene expression in lung cancer. *J Natl Cancer Inst* **81**: 1144–1150

Miyashita T and Reed JC (1992) bcl-2 gene transfer increases relative resistance of S49.1 and WEHI7.2 lymphoid cells to cell death and DNA fragmentation induced by glucocorticoids and multiple chemotherapeutic drugs. *Cancer Res* **52**: 5407–5411

Miyashita T and Reed JC (1993) Bcl-2 oncoprotein blocks chemotherapy-induced apoptosis in a human leukemia cell line. *Blood* **81**: 151–157

Monia BP, Sasmor H, Johnston JF, Freier SM, Lesnik EA, Muller M, Geiger T, Altmann KH, Moser H and Fabbro D (1996) Sequence-specific antitumor activity of a phosphorothioate oligodeoxyribonucleotide targeted to human C-raf kinase supports an antisense mechanism of action in vivo. *Proc Natl Acad Sci USA* **93**: 15481–15484

© Cancer Research Campaign 1998

Ohmori T. Podack ER. Nishio K. Takahashi M. Miyahara Y. Takeda Y. Kubota N. Funayama Y. Ogasawara H. Ohira T. Ohta S and Saijo N (1993) Apoptosis of lung cancer cells caused by some anti-cancer agents (MMC. CPT-11. ADM) is inhibited by bcl-2. *Biochem Biophys Res Commun* **192**: 30–36

Photiou A. Shah P. Leong LK. Moss J and Retsas S (1997) In vitro synergy of paclitaxel (Taxol) and vinorelbine (navelbine) against human melanoma cell lines. *Eur J Cancer* **33**: 463–470

Reeve JG. Xiong J. Morgan J and Bleehen NM (1996) Expression of apoptosis-regulatory genes in lung tumour cell lines: relationship to p53 expression and relevance to acquired drug resistance. *Br J Cancer* **73**: 1193–1200

Skorski T. Nieborowska Skorska M. Wlodarski P. Perrotti D. Hoser G. Kawiak J. Majewski M. Christensen L. Iozzo RV and Calabretta B (1997) Treatment of Philadelphia leukemia in severe combined immunodeficient mice by combination of cyclophosphamide and bcr/abl antisense oligodeoxynucleotides. *J Natl Cancer Inst* **89**: 124–133

Souhami RL and Law K (1990) Longevity in small cell lung cancer. A report to the Lung Cancer Subcommittee of the United Kingdom Coordinating Committee for Cancer Research. *Br J Cancer* **61**: 584–589

Stein CA (1995) Does antisense exist? *Nature Med* **1**: 1119–1121

Stein CA and Cheng YC (1993) Antisense oligonucleotides as therapeutic agents – is the bullet really magical? *Science* **261**: 1004–1012

Stein CA and Krieg AM (1994) Problems in interpretation of data derived from in vitro and in vivo use of antisense oligodeoxynucleotides. *Antisense Res Dev* **4**: 67–69

Strasser A. Harris AW. Jacks T and Cory S (1994) DNA damage can induce apoptosis in proliferating lymphoid cells via p53-independent mechanisms inhibitable by Bcl-2. *Cell* **79**: 329–339

Szczylik C. Skorski T. Nicolaides NC. Manzella L. Malaguarnera L. Venturelli D. Gewirtz AM and Calabretta B (1991) Selective inhibition of leukemia cell proliferation by BCR-ABL antisense oligodeoxynucleotides. *Science* **253**: 562–565

Versantvoort CH. Rhodes T and Twentyman PR (1996) Acceleration of MRP-associated efflux of rhodamine 123 by genistein and related compounds. *Br J Cancer* **74**: 1949–1954

Webb A. Cunningham D. Cotter F. Clarke PA. di Stefano F. Ross P. Corbo M and Dziewanowska Z (1997) BCL-2 antisense therapy in patients with non-Hodgkin's lymphoma. *Lancet* **349**: 1137–1141

Ziegler A. Luedke GH. Fabbro D. Altmann K-H. Stahel RA and Zangemeister-Wittke U (1997) Induction of apoptosis in small-cell lung cancer cells by an antisense oligodeoxynucleotide targeting the bcl-2 coding sequence. *J Natl Cancer Inst* **89**: 1027–1036

© Cancer Research Campaign 1998